# Taurine ameliorates volatile organic compounds-induced cognitive impairment in young rats *via* suppressing oxidative stress, regulating neurotransmitter and activating NMDA receptor

**DOI:** 10.3389/fvets.2022.999040

**Published:** 2022-09-16

**Authors:** Yongchao Gao, Chao Sun, Ting Gao, Zhiyong Liu, Zhao Yang, Hui Deng, Peng Fan, Junhong Gao

**Affiliations:** ^1^Toxicology Research Center, Institute for Hygiene of Ordnance Industry, Xi'an, China; ^2^Xijing Hospital, The Fourth Military Medical University, Xi'an, China

**Keywords:** taurine, volatile organic compounds, cognitive impairment, oxidative stress, neurotransmitter, NMDAR1

## Abstract

Long-term exposure to volatile organic compounds (VOCs) in children leads to intellectual and cognitive impairment. Taurine is an essential nutritional amino acid for children, which can improve neurological development in children. However, the neuroprotective effect of taurine on VOCs-induced cognitive impairment in children remains unclear. The aim of this study was to investigate the neuroprotective effects of taurine on VOCs-induced cognitive impairment in young rats. The rats were nose-only exposed to VOCs for a period of 4 weeks to create a model of cognitive impairment, and 0.5% and 1% taurine in tap water were administered throughout the trial period, respectively. Our results showed that young rats adjusted the recovery of their physiological functions by voluntarily increasing the intake of taurine in tap water when exposed to excessive VOCs by inhalation. In addition, taurine enhanced grasp, shortened the latency period of escape, and improved the learning and memory function of young rats. Moreover, taurine decreased malondialdehyde (MDA), γ-aminobutyric acid (GABA), Aspartate aminotransferase (AST), Alanine aminotransferase (ALT), Urea, Creatinine (CREA) and injury biomarker level, enhanced superoxide dismutase (SOD), reduced glutathione (GSH) and glutamic acid (Glu) activities, up-regulated the protein expression of brain derived neurotrophic factor (BDNF) and N-Methyl-d-aspartate receptor 1 (NMDAR1) in model rats, and in most of cases 1% but not 0.5%, ameliorated the defects induced by VOCs. Collectively, these findings suggested that taurine protected against VOCs-induced cognitive-behavioral impairment in young rats through inhibiting oxidative stress and regulating neurotransmitter homeostasis. In addition, taurine were capable of restoring abilities of learning and memory in young rats exposed to VOCs by activating the N-Methyl-d-aspartate (NMDA) receptor. The findings suggest taurine as a potential novel drug for the treatment of cognitive behavioral disorders in children.

## Introduction

With the wide and extensive application of new building materials and decorative materials, including coatings, adhesives, furniture, flooring, wallpaper, etc., will release a variety of volatile organic compounds (VOCs), which aggravates indoor air pollution. Because of the increasing concentration of indoor chemical compounds and the fact that most people spend the majority of their time inside, indoor air pollution has become a major public health concern (1). In humans, VOC exposure can cause negative health effects, for example, neurological reactions, behaving as weakness, loss of appetite, fatigue, disorientation, and nausea (2). In the VOCs series of compounds, some kinds of VOCs have strong toxicity, serious harm to human health. Benzene series and formaldehyde have been determined as class I carcinogen by the International Center for Research on Cancer (3). Indoor is the main place for human activities, and the indoor environment is closely related to the health of people (4). However, indoor air quality (IAQ) is a common problem in new residential buildings in China (5). With few precautions, inhalation and skin exposure to unhealthy levels of concentration are almost inevitable. VOCs are the main indoor air pollutants, of which benzene, toluene, xylene and formaldehyde are the more common indoor VOCs, including smoking, solvent use, renovation and household products, especially in the presence of new houses and furniture (6). VOCs tests were carried out on more than 7,000 residences in China in the last decade (nearly one-third were newly renovated) and found ubiquitous pollutants exceeding recommended concentrations, including particulate matter, formaldehyde, benzene and other VOCs and molds (7). Human exposure to VOCs includes direct contact through hands and other skin surfaces, as well as inhalation of gases and airborne particulate matter (8).

City dwellers typically spend more than 90% of their time indoors (9). As one of the specially sensitive groups, children are in an important stage of growth and development. They spend more time and volume of breath indoors than adults, and are more vulnerable to pollution than adults due to physiological conditions and other factors (10), so they are more sensitive to indoor environmental pollution. Epidemiological investigations have shown that VOCs exposure can cause neurological symptoms such as headaches and inattention (11). Previous animal studies have shown that VOCs has multi-target organ toxicity and can cause damage to multiple organs and systems in the body. Sub-chronic exposure of low-dose VOCs can damage the body shape and motor function of mice, as well as the learning and memory ability of mice (12). Acute inhalation exposure of high-dose formaldehyde can induce cognitive deficits in mice, causing damage to the hippocampal region and leading to learning and memory disorders (13). Benzenes such as toluene are also well-known neurotoxins (14). The adaptability of infant rats to such as adverse environment is weaker than that of adult rats (15), and the cognitive impairment is particularly serious. Mice's motor function, as well as learning and memory abilities, can be affected by subchronic exposure to low-dose VOCs (16). Recent studies have reported that variable age stages of animals show different amounts of oxidative stress in response to VOCs exposure, and infant rats have weaker adaptability to adverse environment than adults (15). Oxidative damage, altered expression of neurotransmitters and NMDA receptors may be the possible mechanisms of VOCs neurotoxicity (12). How to improve neurotoxicity and cognitive impairment caused by VOCs has become an important problem to be solved urgently. In terms of drug prevention and treatment, neurostimulants and hormone drugs such as amphetamine and dexamethasone have a certain protective effect on cognitive impairment, but their side effects are large and cannot be used as routine preventive drugs (17). Research on how to effectively reduce VOCs damage to brain tissue through dietary approach is of great significance for improving children's intelligence and cognitive ability.

Taurine (2-aminoetaurine), which is an amino acid that may be found in practically all animal tissues, is abundant in the nervous system. It is the only free amino acid second only to Glutamate. Its physiological functions include antioxidant, growth promotion, nervous, cardiovascular, immune and endocrine regulation (18, 19). Taurine has a variety of impacts on the central nervous system: Neuromodulators, neurotrophic agents and neuro protective agents can protect neurons in neuro-related diseases (20), improve fetal brain development (21). It can also significantly improve the cognitive impairment of young rats after prenatal stress (22). In animal studies, taurine has an age-dependent effect on overall cognitive development. A study found that mice in the post-weaning group learned tasks faster than mice in the control group during the first four phases of adulthood (lifetime, pre-weaning, post-weaning, and control) (23). Taurine (dissolved in 1% and 2% tap water, respectively), ameliorates cognitive impairment and inhibits apoptosis of hippocampal neurons exposed to high Glucose in diabetic rats through the NGF-Akt/Bad pathway (24). In this study, the VOCs mixed inhalation exposure method was used to establish a model of cognitive function impairment in young rats. This method was consistent with the exposure environment of indoor VOCs pollution, in reality, and the protective effect of taurine on VOCs-induced cognitive function impairment was evaluated from the aspects of neuro behavior, oxidative stress, brain tissue morphology and related active factors. The underlying molecular mechanisms that may be involved in taurine's improvement of behavioral disorders were elucidated.

## Materials and methods

### Reagents and kits

Taurine (Food grade, Jiangsu Xinrui Biotechnology Co., Ltd. China), Formaldehyde, Benzene, Toluene, Xylene (Analytical purity, Chengdu Cologne Chemical Co., Ltd. China), Malondialdehyde (MDA), Reduced Glutathione (GSH), Superoxide dismutase (SOD), Catalase (CAT), Glutamic acid (Glu) Determination Kit (Nanjing Jiancheng Institute of Biological Engineering, China), Aspartate aminotransferase (AST), Alanine aminotransferase (ALT), Urea, Creatinine (CREA) Determination Kit (Beckman, USA), Rat Glial fibrillary acidic protein (GFAP), Myelin basic protein (MBP), Neurofilament light chain (NF-L), γ-aminobutyric acid (GABA) and Taurine (TAU) ELISA Kit (Shanghai Enzyme-Linked Biotechnology Co., Ltd. China), Anti-BDNF antibody (ab108319) (Abcam, Britain), Anti-NMDAR1 antibody (ab274377) (Abcam, Britain).

### Animals

SD rats were purchased from Sibeifu (Beijing) Biotechnology Co., LTD., with the production license: SCXK (Beijing) 2019-0010. At the beginning of the infection, the animals were 4 weeks old, weighing 89.2 ± 9.1 g, and kept in the barrier system. The license was SYXK (Shaanxi) 2021-008. The ambient temperature was 22°C~26°C, the relative humidity was 40–70%, and the light and shade were alternating with 12 h. The rats fed and drank freely. The experiment was followed the National Institutes of Health guide for the care and use of Laboratory animals (NIH Publications No. 8023, revised 1978) and approved by the Laboratory Animal Management and used Committee of the unit, approval number: IACUC202104.

### Experimental design

Twenty-four healthy young SPF male SD rats were randomly divided into four groups of six rats in each group: control group (control), VOCs model group (VOCs), VOCs+0.5% taurine intervention group (VOCs+0.5% taurine) and VOCs+1%taurine intervention group (VOCs+1% taurine). VOCs, VOCs+0.5% taurine and VOCs+1% taurine groups were exposed to VOCs (Theoretical concentration of 5 mg/m^3^ formaldehyde+5 mg/m^3^ benzene+10 mg/m^3^ toluene+10 mg/m^3^ xylene) mixed liquid aerosol (25) by nose-only inhalation for 4 h/day, 5 day/week for a period of 28 days (4 weeks). The concentrations of formaldehyde and benzene series were dynamically monitored by pump formaldehyde detector and gas chromatography, respectively, to ensure that the actual concentration was consistent with the theoretical concentration. At the same time, the control group inhaled clean air with an inhalation exposure system for 28 days. The VOCs+0.5% taurine and VOCs+1% taurine groups were dissolved in tap water with 0.5% and 1% taurine, respectively. They were given tap water every day from the beginning of modeling to the end of modeling. Control and VOCs model group drank water normally. During the experiment, the body weight of the animals was measured every week, and the drinking volume of each cage (2 rats in 1 cage) was recorded daily by the scale on the drinking bottle, and the daily drinking volume of each rat was obtained after averaging.

### Nose-only inhalation exposure

Four 20-port nose-only inhalation exposure devices are available at the facility (TSE Systems, Germany). Each inhalation exposure system consists of a nose-only inhalation exposure chamber, an aerosol generation system, and a test atmosphere monitor and control system. The test atmosphere was created by aerosolizing the test formulation, a mixture of VOCs in 5% formaldehyde, 5% benzene, 10% toluene, 10% xylene and 70% water with a nebulizer (TSE Systems, Germany). The total air flow was set to produce an air flow of approximately 1 L/min/exposure port. The concentrations of VOCs in the formulations were adjusted to control the aerosol concentrations. To ensure an oxygen concentration of at least 19%, mass flow controllers and a Daco monitoring and control system (TSE Systems, Germany) were used to control the input and exhaust air flows to and from the chamber (26).

The capacity of the nose-only inhalation exposure systems was validated prior to the *in vivo* inhalation exposure investigation. To verify that the concentrations in the aerosols were within 10% of the target concentrations, the parameters of test atmosphere in rat breathing zones were measured for 1 h per day for 3 days (simulated exposure). The concentrations of formaldehyde and benzene series were dynamically monitored using a PGM620 pump-suction formaldehyde detector (RAE, USA) and GC-type gas chromatograph (Shimadzu, Japan).

### Neurobehavioral test

After the 28-day inhalation exposure experiment, the rats were, firstly, tested for their grasping power using the rat and mouse gripping force tester (Shanghai Ruanxin, Inc. China), then the multi-condition behavioral system (TSE Systems, Germany) was used to test the rat's spontaneous activity in the open-field test, and finally the Morris The water maze (Anhui Zhenghua Biology, China) tests the learning and memory abilities of rats.

#### Grip strength test

After the inhalation exposure, the grasping force of each animal was measured. All experiments were conducted in triplicate for technical replication of each animal. The average value was taken as the grasping force value.

#### Open field test

All animals were acclimated in the behavioral testing room for 1 h before the open-field experiment (27). The multi-condition animal behavior system was used for testing, and the animals were placed in the open-field experimental box facing the wall of the experimental box, and after allowing them to adapt for 5 min, the activities of 5 min were recorded. After each animal experiment, the feces in the experimental box were cleaned up, sprayed with alcohol, and dried with a clean towel to continue the subsequent experiment.

#### Morris water maze test

MWM includes place navigation test and spatial probe test, according to Wang's team for specific test methods (12). The experimental period was 7 days. During the experiment, the animals were fed in the behavioral test room to adapt to the experimental environment. The animals were put into the pool from any quadrant on the 1st day of the experiment to swim for 2 min. Positioning and navigation experiment: a total of 5 days (days 2–6), training every day from 2 p.m. to 5 p.m. Before the training, put the platform in quadrant IV, put the animals into the pool from different quadrants facing the pool wall, and record the time when the animals climb onto the platform (escape latency); Space exploration experiment: on the 7th day, remove the platform before the experiment, put the animals into the pool from quadrant II, and record the residence time of the animals in the target quadrant (quadrant IV) and the times of crossing the platform position within 120's.

### Detection of biomarkers of nerve injury and general biochemical parameters in rat serum

After the behavioral test, the rats were put into a CO_2_ anesthesia box, and 40% CO_2_ was introduced for about 2–5 min to achieve the anesthesia effect, and then blood was collected from the abdominal aorta. The collected blood was centrifuged at 3,000 rpm for 10 min, the upper serum was isolated. The contents of GFAP, MBP and NF-L in serum of animals in each group were determined by ELISA test, according to the instructions of rat GFAP, MBP and NF-L ELISA Kit (Shanghai enzyme linked Biotechnology Co., Ltd. China). In addition, the contents of AST, ALT, urea and CREA in serum of animals in each group were determined by automatic biochemical analyser (Beckman, USA).

### Detection of oxidative stress and neurotransmitter level in rat brain tissue

The brain tissues of rats in each group were collected, and normal saline was added at 1:9 to make brain tissue homogenate, centrifuged at 1,000 rpm for 10 min, and the contents of SOD, CAT, GSH, MDA and Glu in the supernatant were determined. The SOD, CAT, GSH, MDA and Glu in the rat brain were detected using commercial kits obtained from Nanjing Jiancheng Bioengineering Research Institute (China) following the manufacturer's instructions. GABA levels in brain tissue were measured using an enzyme-linked immunosorbent assay. The detection was carried out according to the instructions of the rat GABA ELISA kit purchased by Shanghai Enzyme-Linked Biotechnology Co., Ltd. (China).

### Detection of taurine content in rat serum, brain, liver and kidney tissue

In order to investigate the dose-dependent effects of taurine, the serum, brain, liver and kidney tissues of rats in each group were collected to detection of taurine content. Tissue samples were processed by adding normal saline at a ratio of 1:9 to make tissue homogenated, and centrifuged at 1,000 rpm for 10 min to obtain supernatant. The detection method was carried out according to the instruction of taurine (TAU) ELISA kit (Shanghai Enzyme-Linked Biotechnology Co., Ltd. China).

### Histopathological observation

Whole-brain tissue sections of rats in each group (*n* = 3) were stained with H&E and Nissl staining. The brain tissue was fixed with neutral formalin solution and the paraffin embedded tissue was used to make 7 μm paraffin sections. Some sections were stained with hematoxylin and eosin dyes (Solarbio, China) for H&E staining, and the other sections were stained with methyl violet dyes and Nissl differentiation solution (Solarbio, China) for Nissl staining. Finally, the images were captured under a microscope (Leica, Germany) after being fully transparent in anhydrous ethanol to xylene and sealed in a neutral resin to observe morphological changes and analyze the number of living neurons in hippocampus (28).

### Western blot analysis

The hippocampus was isolated from brains of each group which washed by 0.9% cold saline on a cold plate and stored at −80°C for detection, and the tissue was broken by adding lysate containing the protease inhibitor. Centrifuge at 12,000 r/min for 15 min at 4°C, take the supernatant, and use BCA kit for protein quantification. Mix the sample and 5× loading buffer thoroughly at a ratio of 1:4, and boil in a water bath at 100°C for 5 min. NMDAR1 (99 kDa), GAPDH (36 kDa), and BDNF (13 kDa) were separated by 15% SDS-PAGE electrophoresis, electrotransferred to 0.22 μm PVDF membrane, and blocked in 5% skim milk at room temperature for 2 h. The blocked membranes were incubated with primary antibody at a dilution of 1:2000 at 4°C overnight. Then the membranes were washed three times each in TBST buffer (TBS buffer containing 0.5% Tween-20) and incubated with 1:4000 diluted horseradish peroxidase-labeled secondary antibody at room temperature for 1 h.

After washing with TBST, the membrane was developed with ECL. Grayscale analysis was performed after taking pictures with a protein gel imaging system. Standard control was performed with GAPDH as an internal referred.

### Statistical analysis

All data were subject to normal distribution and homogeneous variance. The MWM data were analyzed by repeated measurement analysis of variance. Significance of the other data was determined with one-way analysis of variance (ANOVA) followed by an LSD test for *post hoc* multiple comparisons. Inspection level α = 0.05.

## Results

### Effects of taurine on body weight and water intake of rats exposed to VOCs

**Figure 1:**
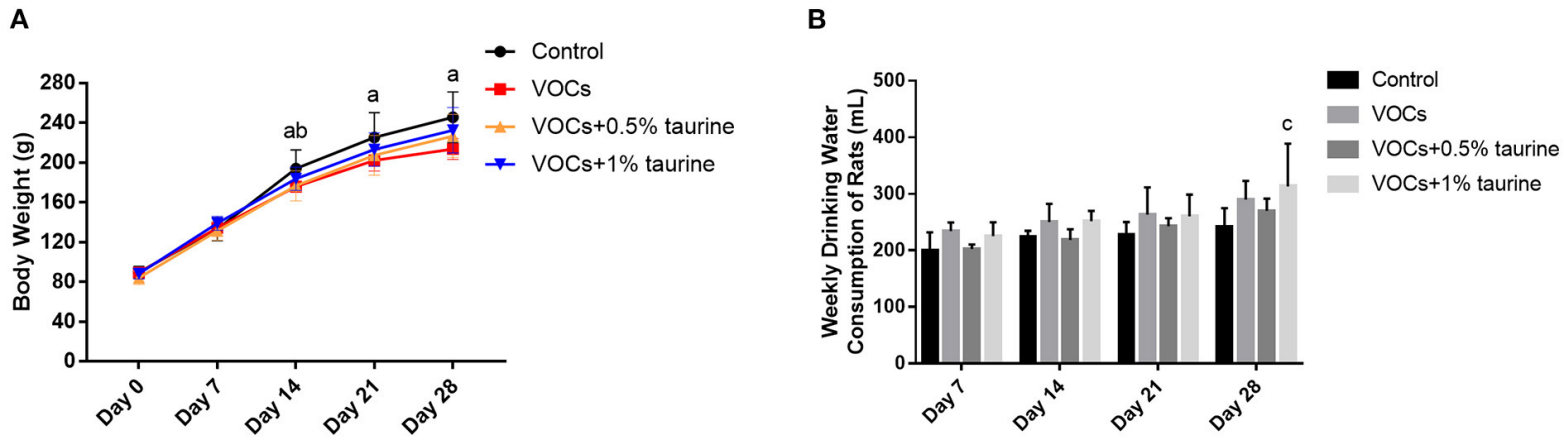
Effects of taurine on body weight and water intake of rats exposed to VOCs. **(A)** The body weight of the rats in each group during the experiment. **(B)** The water intake of the rats in each group during the experiment. Data are expressed as mean ± SD. (*n* = 6). a: *P* < 0.05, VOCs group vs. control group. b: *P* < 0.05, VOCs+0.5% taurine group vs. control group. c: *P* < 0.05, VOCs+1% taurine group vs. control group. d: *P* < 0.05, VOCs+0.5% taurine group vs. VOCs group. e: *P* < 0.05 VOCs+1% taurine group vs. VOCs group.

During inhalation exposure, the activities and diet of rats in each dose group were normal without death. The body weight and water intake of rats in each group during the experiment were shown in Figure 1. The results showed no significant difference in body weight between day 0 and 7 (*P* > 0.05). In day 14, the significantly lower body weight of rats was observed in VOCs group and VOCs+0.5% taurine group than that in control group (*P* < 0.05), whereas there was no significant difference between VOCs+1% taurine and control group (*P* > 0.05). On day 21 and 28, only the body weight of rats in VOCs group showed significant difference compared with control group (*P* < 0.05), but no significant difference was observed among other groups (*P* > 0.05, Figure 1A). From the 1st week to the 3rd week of the experiment, there was no difference in the water consumption of the rats in each group (*P* > 0.05), but in the 4th week, the water consumption of the rats in the VOCs+1% taurine group was higher than that in the control group (*P* < 0.05, Figure 1B).

### Effects of taurine on grasping strength, autonomous activity and exploration behavior of rats exposed to VOCs

**Figure 2:**
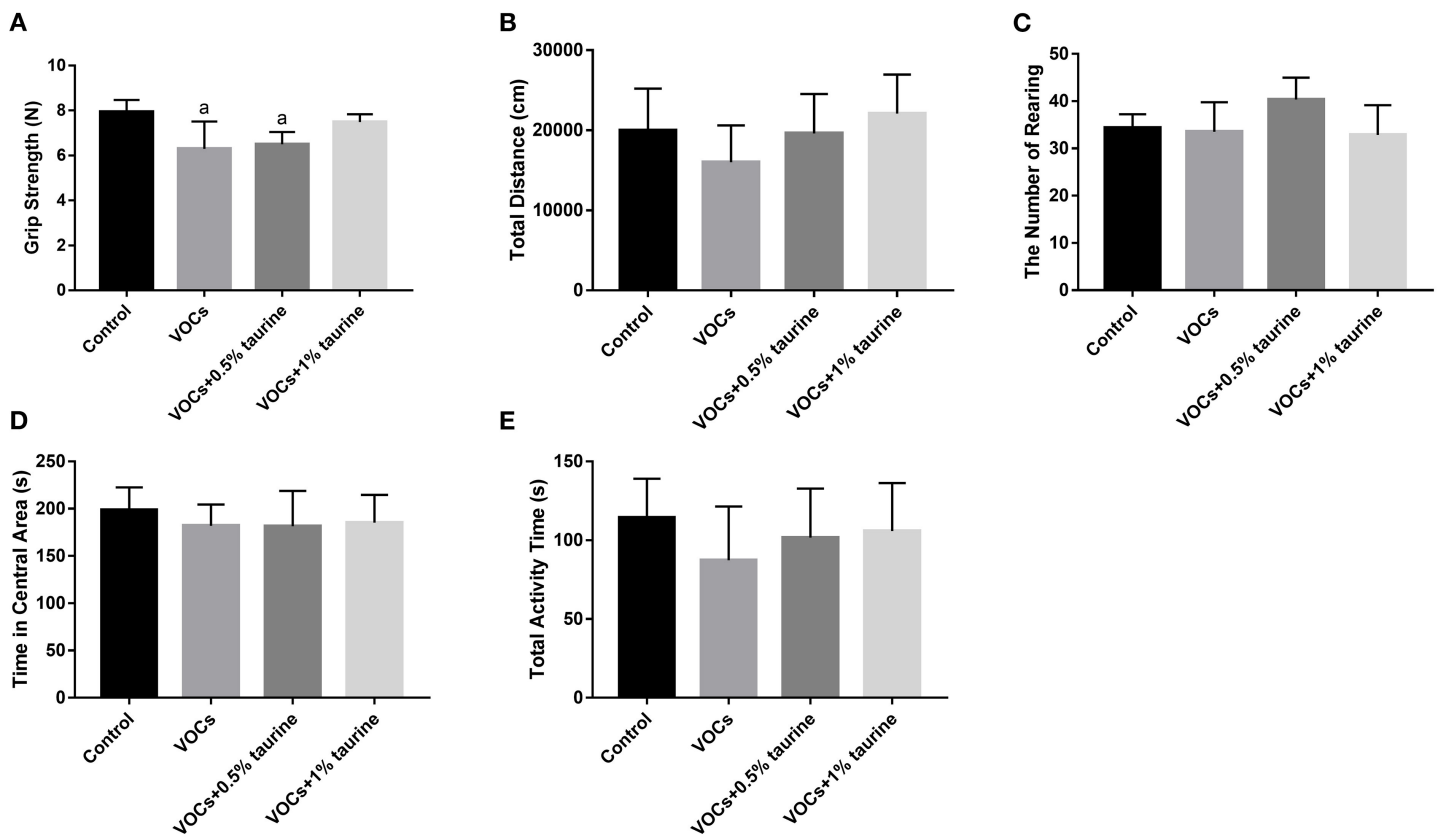
Effects of taurine on grasping strength, autonomous activity and exploration behavior of rats exposed to VOCs. **(A)** Grasping strength. **(B)** Total distance. **(C)** The number of rearing. **(D)** Time in central area. **(E)** Total activity time. Data are expressed as mean ± SD. (*n* = 6). a: *P* < 0.05 vs. control group. b: *P* < 0.05 vs. VOCs group. c: *P* < 0.05 vs. VOCs+0.5% taurine group.

After VOCs exposure, the rats in each group were tested for grasping strength (Figure 2A). The results showed that the grasping strength of rats in VOCs and VOCs+0.5% taurine group decreased significantly, compared with control group (*P* < 0.05). It showed no significant difference in grasping force between the VOCs+1% taurine group and control group (*P* > 0.05). The results demonstrated that taurine reversed grasping strength of rats exposed to VOCs.

To figure out the effects of taurine on autonomous activity and exploring behavior in VOC-exposed rats, we did an open field test. As a result, no significant difference was showed in total distance, total activity time, time in central area and number of rearing of rats in each group (*P* > 0.05, Figures 2B–E). Compared with control group, the total distance and activity time of rats in VOCs group showed a downward trend, while taurine slightly recovered autonomous activity and exploration behavior.

### Effects of taurine on learning and memory ability of rats exposed to VOCs

**Figure 3:**
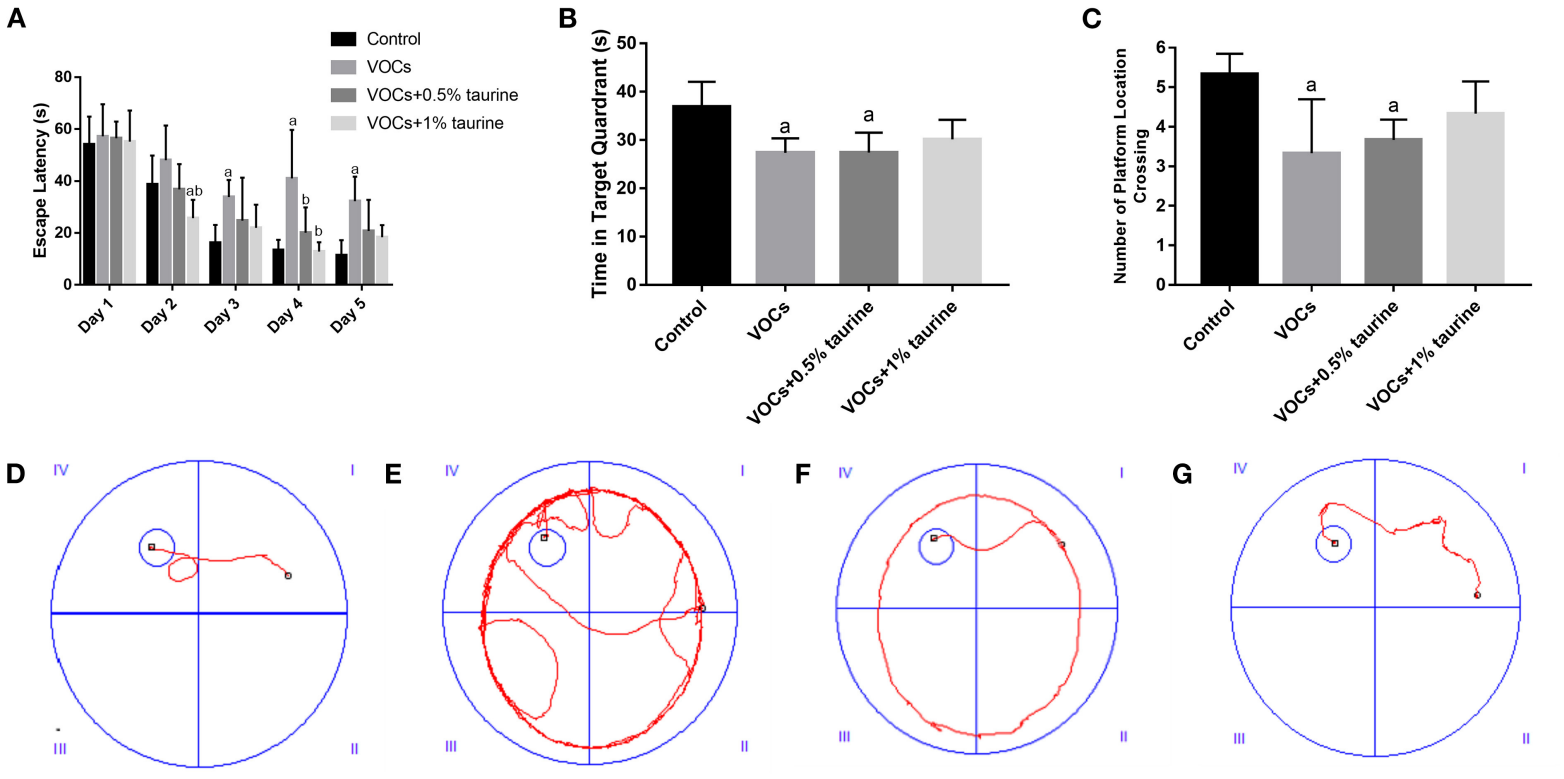
Effects of taurine on learning and memory of rats exposed to VOCs. **(A)** Escape latency. **(B)** Time in target quadrant. **(C)** Number of platform location crossing. **(D–G)** Movement tracks of rats in control group, VOCs group, VOCs+0.5% taurine group and VOCs+1% taurine group. Data are expressed as mean ± SD. (*n* = 6). a: *P* < 0.05 vs. control group. b: *P* < 0.05 vs. VOCs group. c: *P* < 0.05 vs. VOCs+0.5% taurine group.

In the place navigation test, through repeated measurement analysis of variance of escape latency data, the results show that the escape latency has a downward trend with the experimental time, and the role of time factors does not vary with different groups, and the escape latency of each group is not the same as a whole (Figure 3A). The results according to one-way ANOVA indicated that there was no significant difference in the escape latency of rats in each group on the 1st day of the test (*P* > 0.05). On the 2nd day, the escape latency of the VOCs+1% taurine group was significantly lower than that of the control and VOCs groups (*P* < 0.05). On the 3rd day, the escape latency of rats in VOCs group was significantly higher than that in control group (*P* < 0.05), but no significant difference was shown among the other three groups (*P* > 0.05). On the fourth day, VOCs group showed the significantly higher escape latency of rats than that in control, VOCs+0.5% taurine and VOCs+1% taurine groups (*P* < 0.05), and there was no significant difference among the other three groups (*P* > 0.05). On the 5th day, the escape latency of rats in VOCs group was significantly higher than that in control group (*P* < 0.05). There was no significant difference among the other three groups (*P* > 0.05).

The results of the spatial probe test were shown in Figures 3B,C. The time in target quadrant and the number of platform location crossing of VOCs group and VOCs+0.5% taurine group were significantly lower than that of the control group (*P* < 0.05). There was no significant difference between the VOCs+1% taurine group and control group (*P* > 0.05).

It showed the movement trajectories of rats in each group (Figures 3D–G). The control and VOCs+1% taurine groups have similar movement trajectories, and the rats can quickly locate the platform position. Compared with control group, the trajectories of VOCs and VOCs+0.5% taurine groups were significantly prolonged.

### Effects of taurine on biomarkers of serum nerve injury and general biochemical parameters of rats exposed to VOCs

**Figure 4:**
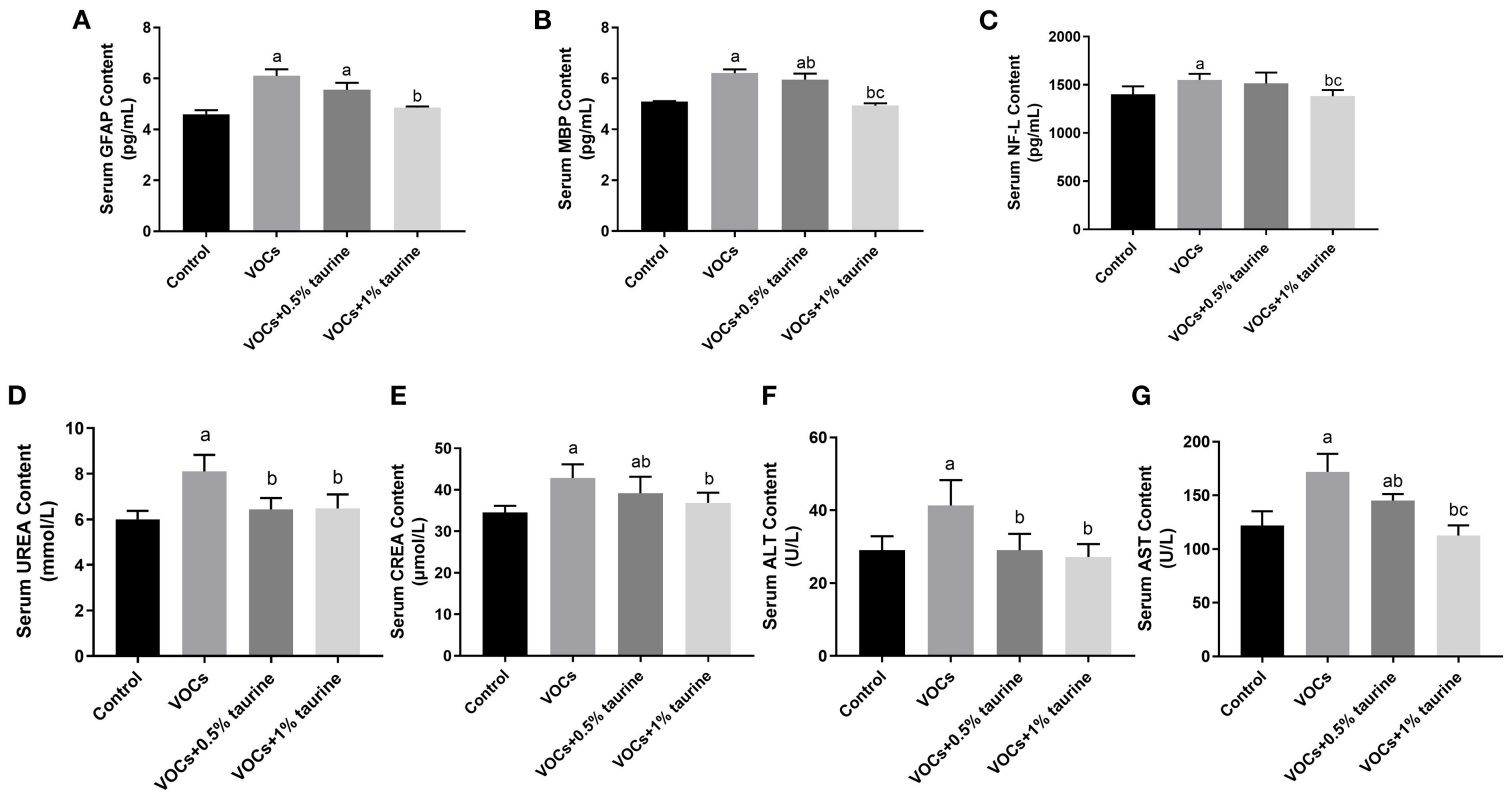
Effects of taurine on biomarkers of serum nerve injury and general biochemical parameters caused by VOCs in rats. **(A)** Contents of GFAP in rat serum. **(B)** Contents of MBP in rat serum. **(C)** Contents of NF-L in rat serum. **(D)** Contents of UREA in rat serum. **(E)** Contents of CREA in rat serum. **(F)** Contents of ALT in rat serum. **(G)** Contents of AST in rat serum. Data are expressed as mean ± SD. (*n* = 6). a: *P* < 0.05 vs. control group. b: *P* < 0.05 vs. VOCs group. c: *P* < 0.05 vs. VOCs+0.5% taurine group.

In Figures 4A–C, the contents of GFAP, MBP, and NF-L in VOCs group were significantly higher than those in control group (*P* < 0.05). There was no difference in the contents of GFAP and NF-L between VOCs group and VOCs+0.5% taurine group (*P* > 0.05). The contents of GFAP, MBP, and NF-L in serum of the VOCs+1% taurine group were not different from those in control group (*P* > 0.05), but lower than those in VOCs group (*P* < 0.05). Furthermore, we tested serum biochemical parameters that reflect liver and kidney function. The results showed that compared with control group, the serum urea, CREA, ALT and AST of VOCs group were significantly increased (*P* < 0.05, Figures 4D–G). The urea and ALT content in serum of rats in VOCs+0.5% taurine and VOCs+1% taurine groups were lower than that in VOCs group (*P* < 0.05, Figures 4D,F), and there was no significant difference compared to the control group (*P* > 0.05). Although the CREA and AST content of VOCs+0.5% taurine and VOCs+1% taurine groups were lower than that of VOCs group (*P* < 0.05), only VOCs+1% taurine group had no significant difference with the control group (*P* > 0.05, Figures 4E,G).

### Effects of taurine on oxidative stress level in brain tissue of rats exposed to VOCs

**Figure 5:**
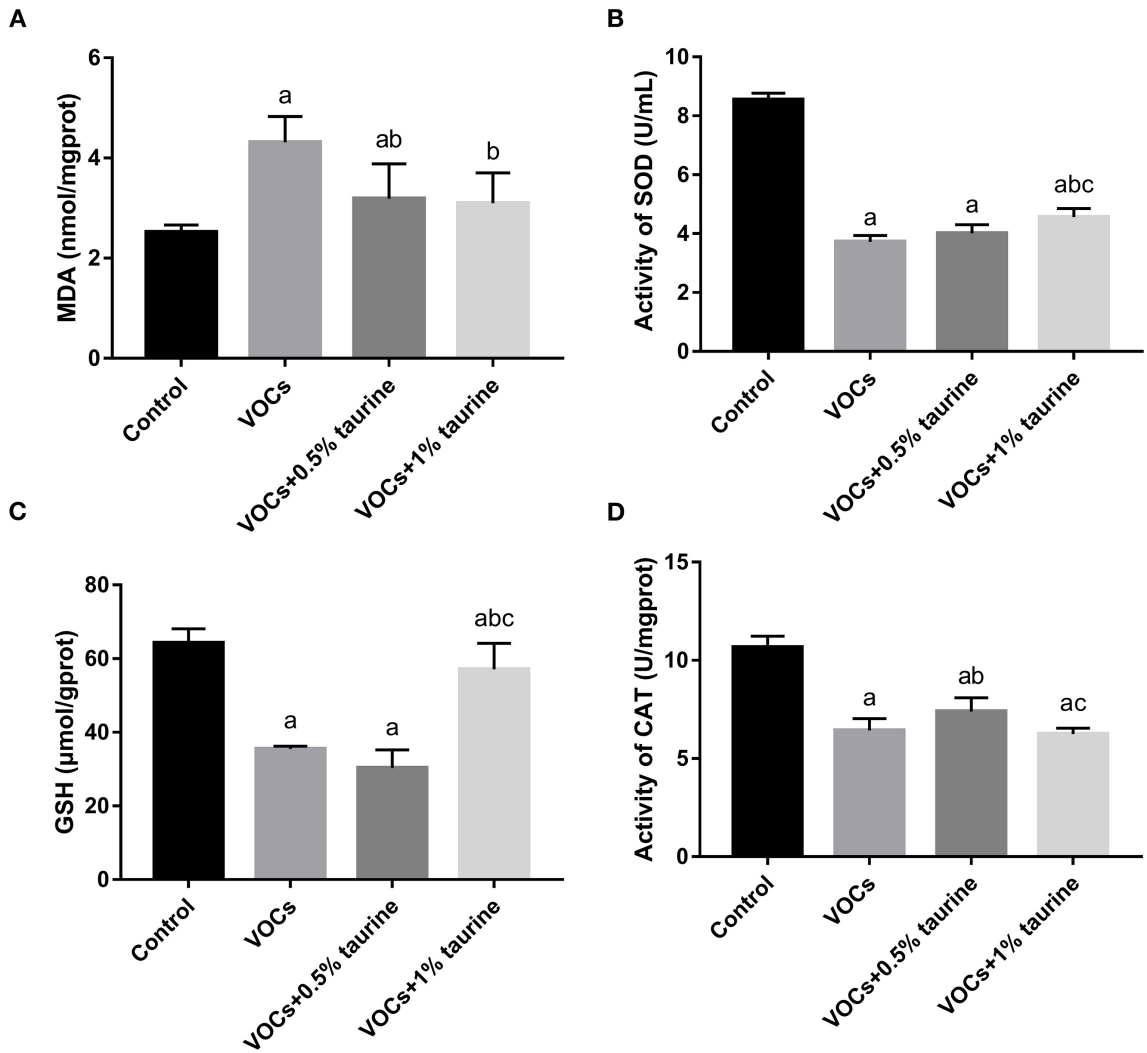
Effects of taurine on oxidative stress level in brain tissue of rats exposed to VOCs. **(A)** MDA content in brain tissue. **(B)** SOD activity in brain tissue. **(C)** GSH content in brain tissue. **(D)** CAT activity in brain tissue. Data are expressed as mean ± SD (*n* = 6). a: *P* < 0.05 vs. control group. b: *P* < 0.05 vs. VOCs group. c: *P* < 0.05 vs. VOCs+0.5% taurine group.

To investigate whether taurine mitigated VOCs-induced lipid peroxidation damage, the antioxidant enzymes SOD, CAT, GSH and lipid peroxidation product MDA in the antioxidant system were measured. The results were shown in Figure 5. Compared with control group, MDA was increased in VOCs group, while SOD, GSH and CAT activities were decreased (*P* < 0.05). The MDA content in brain tissues of rats in VOCs+0.5% taurine and VOCs+1% taurine groups was lower than that in VOCs group (*P* < 0.05), and there was no significant difference between VOCs+1% taurine and control groups (*P* > 0.05, Figure 5A). Rats in the VOCs+1% taurine group had higher SOD activity and GSH concentration in brain tissue than rats in the VOCs group (*P* < 0.05, Figures 5B,C). However, there was no significant difference in CAT activity between 1% taurine group and the VOCs group (*P* > 0.05, Figure 5D).

### Effects of taurine on neurotransmitters in brain tissue of rats exposed to VOCs

**Figure 6:**
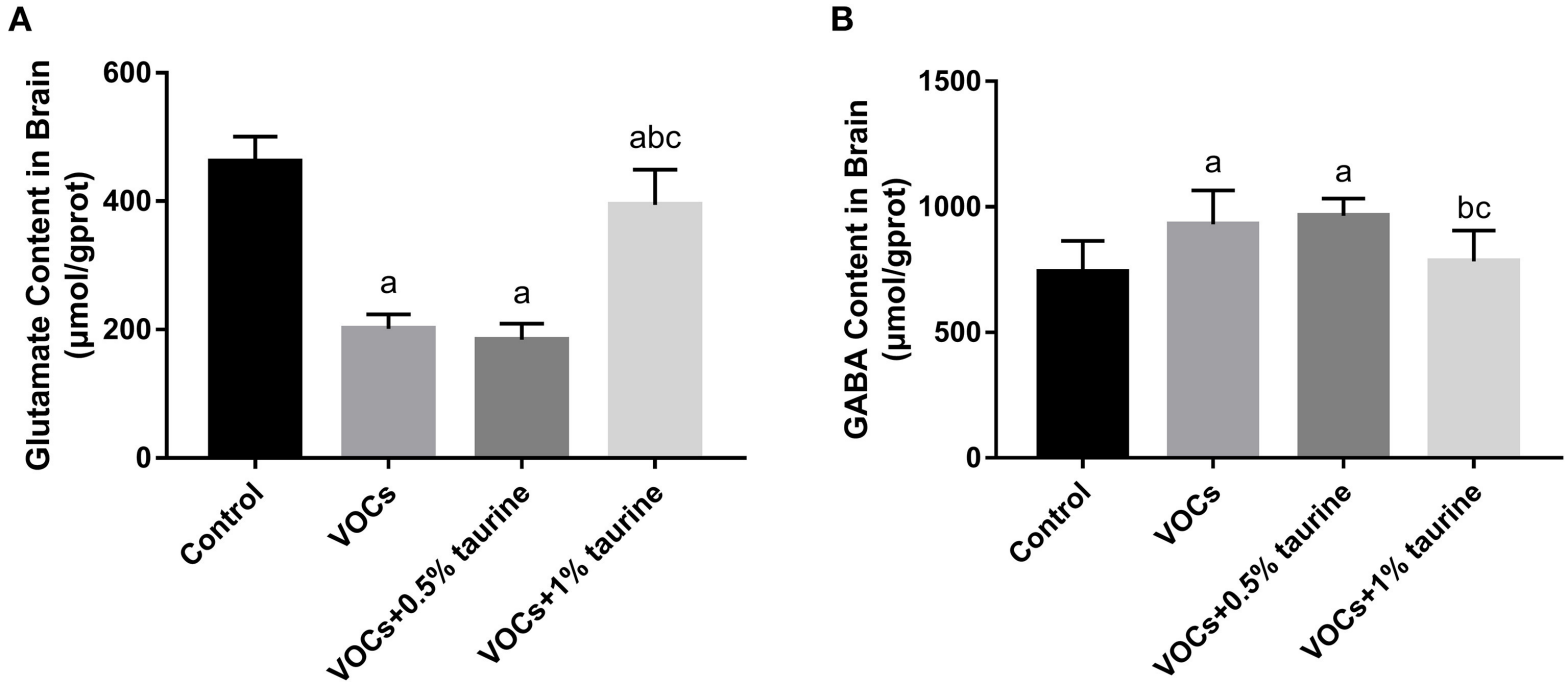
Effects of taurine on neurotransmitters in brain tissue of rats exposed to VOCs. **(A)** Glutamate content in brain tissue. **(B)** γ - aminobutyric acid content in brain tissue. Data are expressed as mean ± SD. (*n* = 6). a: *P* < 0.05 vs. control group. b: *P* < 0.05 vs. VOCs group. c: *P* < 0.05 vs. VOCs+0.5% taurine group.

The contents of Glu and GABA in rat brain tissues of each group were shown in Figure 6. Glu was found to be considerably lower in the VOCs and VOCs+0.5% taurine groups compared to the control group, but GABA concentration was significantly higher (*P* < 0.05). Glu was significant higher in the VOCs+1% taurine group compared to VOCs and VOCs+0.5% taurine groups (*P* < 0.05), and GABA was lower in the VOCs+1% taurine group (*P* < 0.05), but there was no significant difference with control group (*P* > 0.05).

### Changes of taurine content in the serum, brain, liver, and kidney tissue of rats exposed to VOCs

**Figure 7:**
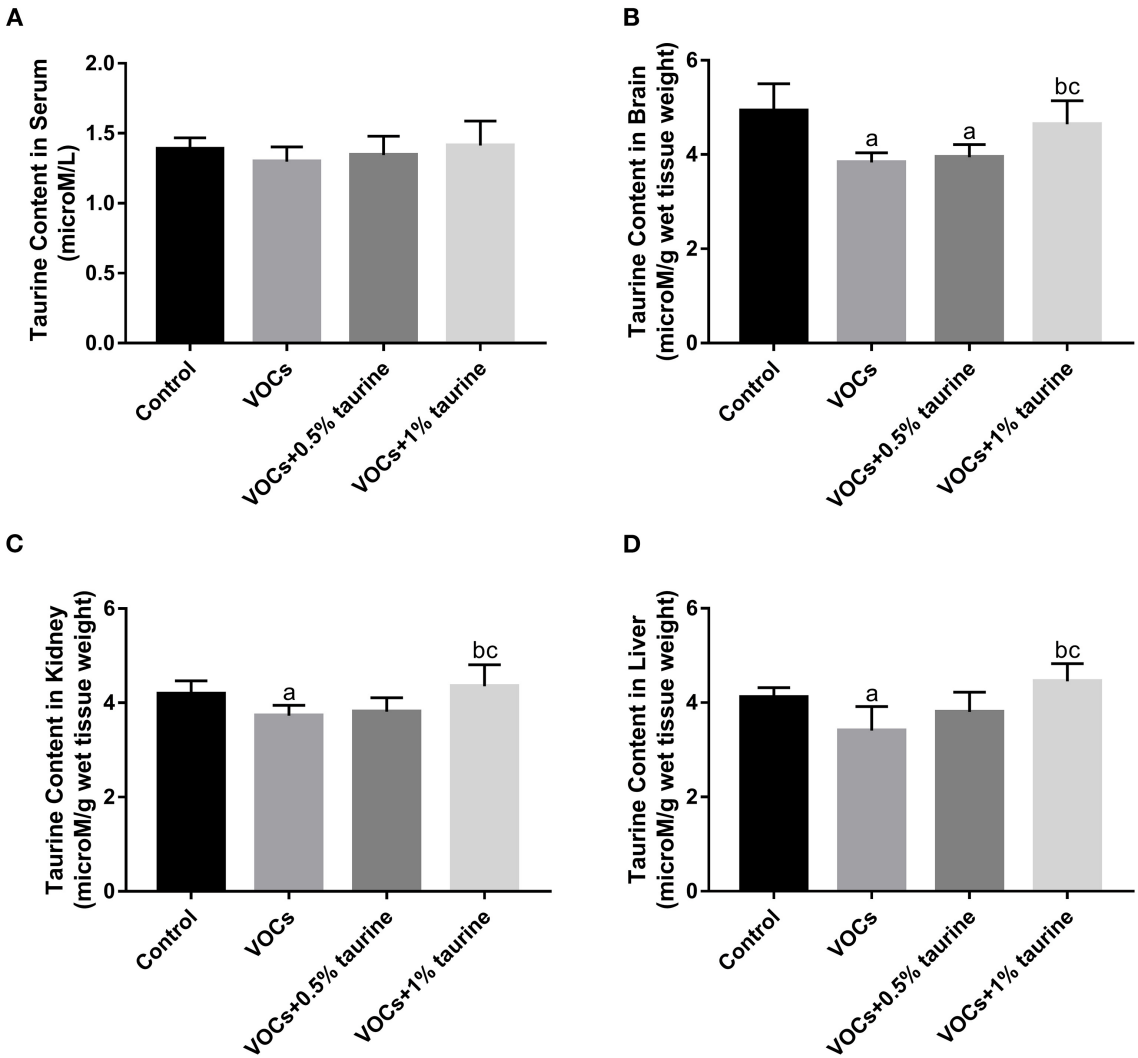
Changes of taurine content in the serum, brain, liver and kidney tissue of rats exposed to VOCs. **(A)** Taurine content in rat serum. **(B)** Taurine content in rat brain. **(C)** Taurine content in rat kidney. **(D)** Taurine content in rat liver. Data are expressed as mean ± SD. (*n* = 6). a: *P* < 0.05 vs. control group. b: *P* < 0.05 vs. VOCs group. c: *P* < 0.05 vs. VOCs+0.5% taurine group.

We further examined taurine levels in serum, brain, liver and kidney tissues to investigate the dose-dependent effects of taurine. The results showed that there was no significant difference in serum taurine content of each group (*P* > 0.05, Figure 7A). However, taurine content in brain, liver and kidney of VOCs group was significantly lower than that of control group. Compared with VOCs and VOCs+0.5% taurine group, taurine content in these tissues was significantly increased in VOCs+1% taurine group (*P* < 0.05, Figures 7B–D).

### Effects of taurine on the hippocampus of rats exposed to VOCs

**Figure 8:**
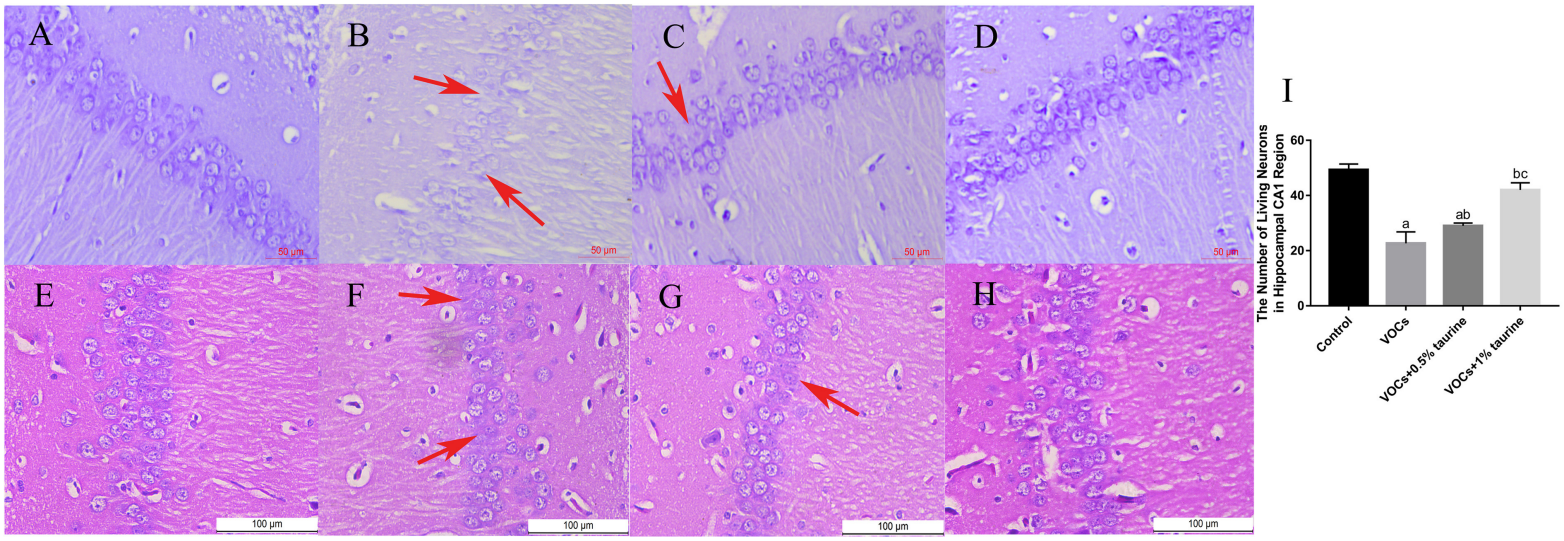
Effects of taurine on the hippocampus of rats exposed to VOCs. **(A)** control group (Nissl staining, ×400). **(B)** VOCs group (Nissl staining, ×400). **(C)** VOCs+0.5% taurine group (Nissl staining, ×400). **(D)** VOCs+1% taurine group (Nissl staining, ×400). **(E)** control group (H&E, ×400). **(F)** VOCs group (H&E, ×400). **(G)** VOCs+0.5% taurine group (H&E, ×400). **(H)** VOCs+1% taurine group (H&E, ×400). **(I)** The number of living neurons in hippocampal CA1 region. Data are expressed as mean ± SD. (*n* = 3). a: *P* < 0.05 vs. control group. b: *P* < 0.05 vs. VOCs group. c: *P* < 0.05 vs. VOCs+0.5% taurine group. The red arrow indicates damaged neuronal cells.

The effects of taurine on VOCs-induced hippocampal neuron injury in rats were investigated by H&E and Nissl staining. Nissl staining showed that neurons in the hippocampus of control group (Figure 8A) were neatly arranged. In VOCs group (Figure 8B), nerve cells in the hippocampal area were scattered and stained slightly. After 0.5% and 1% taurine intervention (Figures 8C,D), the neurons showed darker staining. H&E staining showed regular arrangement of cells in the hippocampus of rats in control group (Figure 8E). The number of neurons in the hippocampus of rats in VOCs group (Figure 8F) decreased and arranged irregularly. Taurine intervention improved hippocampal nerve injury in VOCs+0.5%taurine and VOCs+1%taurine groups (Figures 8G,H). Compared with VOCs group, the number of viable neurons in the hippocampal CA1 region of rats in VOCs+1%taurine group (Figure 8I) (*P* < 0.05) increased with regular arrangement.

### Effects of taurine on BDNF and NMDAR1 protein expression in hippocampus of rats exposed to VOCs

**Figure 9:**
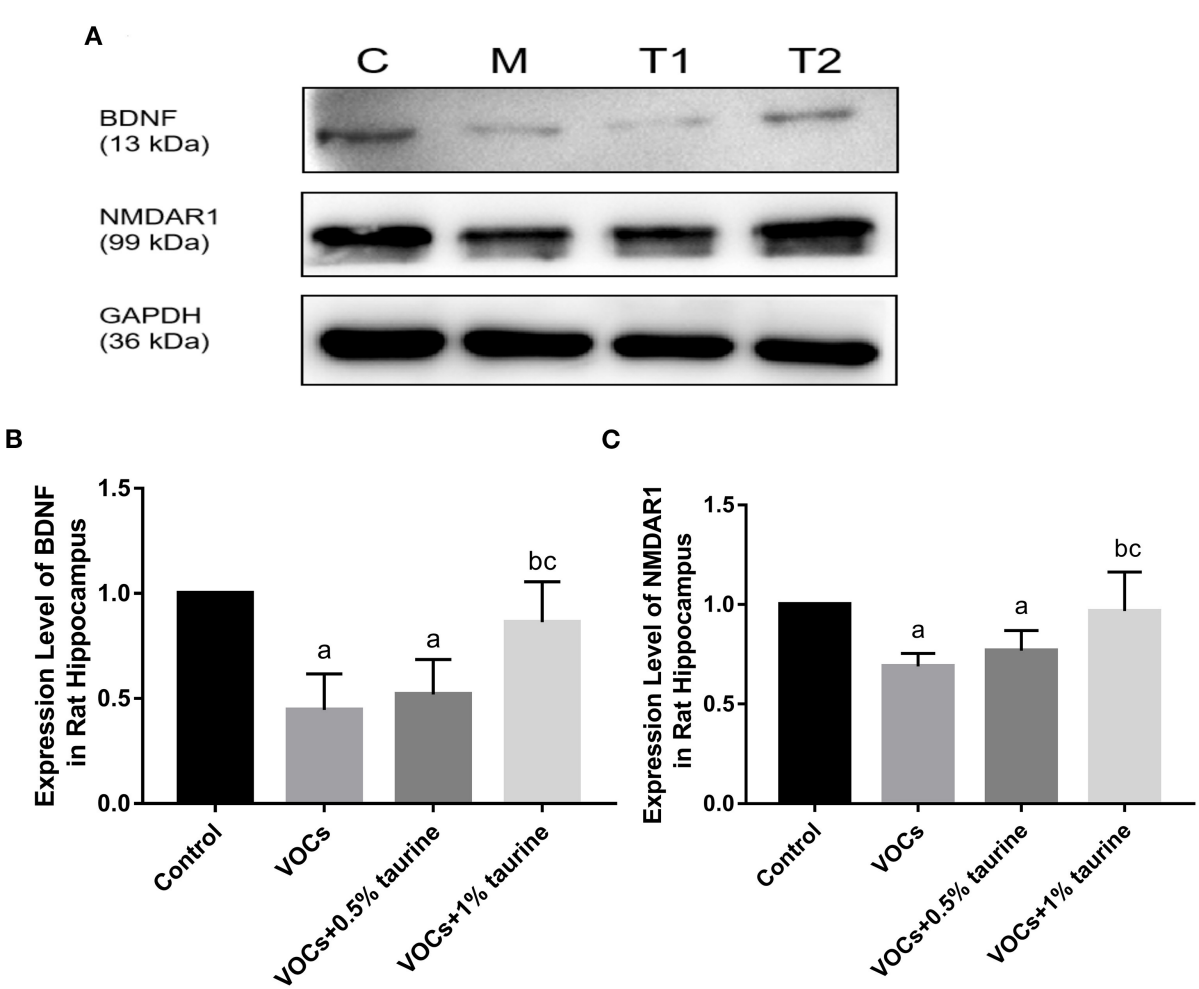
Effects of taurine on BDNF and NMDAR1 protein expression in hippocampus of rats exposed to VOCs. **(A)** Western blot analyses of BDNF and NMDAR1 proteins. GAPDH served as a loading control. **(B)** Western blots analysis showing the effect of taurine on BDNF protein expression level. **(C)** Western blots analysis showing the effect of taurine on NMDAR1 protein expression level. Data are expressed as mean ± SD. (*n* = 3). a: *P* < 0.05 vs. control group. b: *P* < 0.05 vs. VOCs group. c: *P* < 0.05 vs. VOCs+0.5% taurine group.

The protein expressions of BDNF and NMDA receptor were evaluated using the western blotting technique to investigate the protective effects of taurine. Compared to the control group, BDNF expression was shown to be significantly lower in the VOCs group (*P* < 0.05, Figure 9). Treatment with 1% taurine significantly upregulated BDNF protein expression in VOCs-exposed rats (*P* < 0.05), while 0.5% taurine did not (*P* > 0.05, Figure 9B). The NMDAR1 protein expression level was found significantly decreased in VOCs and VOCs+0.5% taurine group (*P* < 0.05). Following 1% taurine treatment, there was a significant activation of NMDAR1 protein expression compared with the VOCs group (*P* < 0.05, Figure 9C).

## Discussion

Taurine has antioxidant and neuroprotective properties (29, 30). Dietary supplementation of taurine has been recognized to improve neurological abnormalities and promote health in infants and children (31). It is particularly prevalent in the developing brain, where it regulates neural progenitor cell proliferation and synaptic formation throughout fetal and neonatal development (32). In this study, we used the formaldehyde, benzene, toluene, xylene mixture of liquid aerosols acting inhaled infected were cognitie impairment model is established, from the nerve behavior, nerve damage markers, oxidative stress and neurotransmitter protein expression etc. Comprehensive study of the taurine to improve the effectiveness of VOCs to her pups cognitie impairment. And we found that taurine protected against VOCs-induced cognitive-behavioral impairment in young rats through inhibiting oxidative stress and regulating neurotransmitter homeostasis. In addition, taurine were capable of restoring abilities of learning and memory in young rats exposed to VOCs by activating the NMDA receptor. This study confirmed the potential protective effect of taurine on VOCs-induced cognitive impairment in young rats, which is of great significance for taurine to improve children's intelligence and cognitive ability through dietary approach.

Animal body weight, food intake, and water consumption are all critical indicators of bodily system toxicity. The weight and food intake of rats in the model group dropped in varied degrees from 2 to 4 weeks after exposure, resulting in delayed weight gain, as compared to the blank control group. However, as compared to the blank control group, the 1% taurine intervention group's weight and food intake remained unchanged. We found that at week 4, the water intake of the 1% taurine intervention group was slightly higher than that of the control group. It was considered that the body could adjust its physiological function recovery by increasing the intake of taurine after exposure to VOCs and formaldehyde mixed inhalation.

Grasp control is closely related to the central nervous system, and is often used to study the neuromuscular function of rodents. Grasping change is an important evidence of motor neurotoxicity. Some scholars have found that subchronic low-dose VOCs exposure damages neuromuscular function in mice (12), which is consistent with our findings. In this experiment, the grasping force of rats in the model group was considerably lower than that of rats in the control group, showing that rats' neuromuscular function was harmed by subacute exposure to VOCs. However, the high-dose taurine intervention group and the control group had the same gripping force, indicating that a 1% taurine intervention can effectively restore the neuromuscular function loss caused by VOCs in rats. In our further study on spontaneous activity and the exploratory behavior, we found that activity time and distance of young rats in the model group were reduced to some extent compared with 1% taurine intervention group and control group. The results indicated that taurine could reverse spontaneous activity and exploration behavior in VOCs damaged rats, but the improvement effect was not significant. At present, a study conducted Morris water maze behavioral test on VOCs modeled animals and found that the escape latency of mice significantly increased and the residence time in the target quadrant decreased after acute or long-term inhalation of VOCs (12). The Morris Water Maze results of this study show that: Model group rats time find the platform, through the platform of the times, stay time in the target quadrant are lower than the control group, shows that VOCs subacute exposure affects the ability of learning and memory of rats, the water intake of taurine intervene and found that 1% taurine intervention group of rats and control rats trajectory is roughly same, can quickly find the platform position. In general, through the analysis of behavioral test results, taurine has a significant improvement effect on VOCs-induced cognitive behavioral damage in young rats.

Serum GFAP, MBP and NF-L are important biomarkers of nervous system injury, which are often used to determine the degree of nervous system injury. GFAP is an intermediate filament involved in astrocyte cytoskeleton construction and is also a central nervous expression protein (33). MBP is the main protein of myelin in central nervous system and is involved in myelin structural proteins (34). NF-L is mainly expressed in axon white matter and is the main component of cytoskeleton. Under normal physiological conditions, these three substances mainly exist in the central nervous system (35). When the nervous system is damaged, GFAP, MBP and NF-L can cross the blood-brain barrier and enter the blood, resulting in increased blood content. In this study, the levels of GFAP, MBP, and NF-L in the serum of rats in the VOCs model group were higher than in the control group, demonstrating that high-dose VOC exposure can cause nervous system injury in young rats. Meanwhile, we found that the abnormally elevated biomarkers of nerve damage in serum of rats in the 1% taurine intervention group could be restored to the normal level. However, the specific mechanism is unclear and needs further study. It has been reported that taurine has liver and kidney protective effects against the harmful effects of a variety of exogenous substances (36, 37). Meantime, VOCs may have toxicity not only in brain but in many other tissues, especially liver and kidney. Therefore, through further check general blood biochemical parameters, we found that the rats in the VOCs+1% taurine group showed significant recovery in serum AST, ALT, UREA and CREA compared with those in the VOCs model group, but not obvious by 0.5% taurine. We speculate that the effect of taurine in peripheral may partly contribute to the action of taurine against brain damage.

Taurine is a key modulator of homeostasis that has a number of roles in providing protection against oxidative stress. Taurine has been demonstrated to protect cultured cells, organs, and mammals from the harmful effects of oxidative stress caused by a wide range of chemicals, such as Arsenic, carbon tetrachloride, Nitrogen content, arsenide, Endosulfan, cisplatin, adriamycin, Streptozotocin, Bisphenol A, etc. Harmful consequences of oxidative stress (38, 39). According to *in vitro* and *in vivo* studies, taurine's neuroprotective potential is due to its antioxidant action in the brain (40). Studies on the neuroprotective mechanism of taurine mainly focus on the activities of acetylcholinesterase and antioxidant enzyme, as well as scavenging oxygen-free radicals and enhancing antioxidant stress ability by preventing the increase of hydrogen peroxide and lipid peroxide levels in animal brain tissues caused by toxic and harmful substances (29, 41, 42). Therefore, we assessed the effect of taurine on VOCs-induced oxidative stress levels. The results found that exposure to MDA content in rats tissue levels of VOCs, endogenous antioxidant GSH content is reduced, this may be due to too many free radicals can't get the enzymes GSH antioxidant substances removal, cause lipid peroxidation, and thus the increase of lipid peroxidation products MDA, lipid peroxidation damage, Taurine treatment significantly prevented oxidative damage to the brain. Taurine was found to considerably reduce the lipid peroxidation damage caused by VOCs in this investigation. Taurine also enhanced the involvement of the antioxidant enzyme SOD in an attempt to combat VOCs-induced oxidative stress, but the effect was not obvious. In addition, histopathology showed that taurine significantly hindered the progression of brain tissue injury, and the effect was most obvious in the 1% taurine intervention group. This may be because the resistance of brain tissue to morphological injury is related to the restoration of antioxidant system.

Oxidative stress subsequently enhances proinflammatory factor release through activation of B cell activator (NF-κB pathway). Changes in the release of neurotransmitters such as Glutamate, neuropeptides, and growth factors are caused by high amounts of pro-inflammatory cytokines, resulting in neuroanxiety and depression (43). BDNF is an important neurotrophic factor that promotes neuronal development, differentiation, survival, and the creation of long-term memories. There is a large amount of evidence for the role of BDNF in the pathogenesis of behavioral disorders (44). It has been proved that by stimulating the PKA-CREB-BDNF signaling pathway *in vitro*, taurine can accelerate neural stem cell differentiation into neurons, raise the ratio of neurons to glial cells, and inhibit gliosis (45). The endocrine system and cerebral development are disrupted by hexabromocyclododecanes (HBCDs), which impairs cognitive function further. Taurine increases protein expression of BDNF and NGF, Significantly improved cognitive impairment caused by HBCDs in developing rats (46). This study also found that 1% taurine treatment can significantly improve VOCs-induced cognitive impairment in young rats by upregulating the protein expression of BDNF. In this VOCs model, we found that the endogenous taurine content in the brain, liver and kidney tissue were significantly reduced. Results showed that the taurine content in the brain, liver and kidney tissue was increased obviously after exogenous 1% taurine supplementation, and with the increase of taurine level, the taurine content in tissues increased in a dose-dependent effect. Therefore, it is believed that 1% taurine treatment may recover the change in taurine content in the brain, liver and kidney tissues and ameliorate the defects induced by VOCs, but not enough by 0.5%.

Central nervous system toxicity usually occurs in an imbalance between excitatory and inhibitory neurotransmitters. Neurotransmitters act as signaling molecules that carry signals from neurons to target cells and regulate a variety of biological processes and behaviors. Glu and GABA are typical neurotransmitters that are important for learning and memory (47). Past studies showed that a low Glu/GABA ratio can cause learning and memory problems (48, 49). The destruction of Glu and GABA balance may be an important pathway for impaired learning and memory (50). In this study, Glu content decreased and GABA content increased in rats brain tissue after VOCs exposure, suggesting that neurotransmitter homeostasis was disrupted and thus impaired learning and memory. Taurine significantly improved the VOCs-induced imbalance of Glu and GABA, suggesting that taurine could play a neuroprotective role by affecting the balance of Glu and GABA. The role of taurine in the central nervous system is largely determined by complex interactions in the Glu/GABA system and NMDA receptors (51). In vertebrate central nerves, ionic Glutamate receptors (iGluRs) are ligand-gated ion channels that mediate most excitatory neurotransmission. The key drivers of synaptic plasticity are NMDA receptors, which are major members of the iGluR family. They are commonly recognized as the main cellular matrix for learning and memory (52). NMDARl is a functional subunit of the NMDA receptor that is involved in synaptic plasticity, memory, and learning. Studies have shown that deletion of the NMDARl gene leads to deficits in social memory in CA3 pyramidal cells (53). Abnormal activation of extracellular NMDA receptor subunits may lead to elevated extracellular Glutamate levels and reduced reuptake, leading to neuronal damage (54). Studies have shown that short-term inhalation of high-dose VOCs mixtures affects learning and memory performance and expression of NMDA receptor subunits in mice (16). Taurine is a weak agonist of NMDA receptor (55). Taurine has been found to protect nervous system and cognitive function by activating GABAA receptors and NMDA receptors in animal models of lead poisoning (56). The expression of NMDAR1 protein in the hippocampus of rats in the VOCs group was shown to be lower in this study, which could be one of the fundamental mechanisms of VOCs affecting learning and memory. Taurine supplementation can considerably reduce the VOCs-induced reduction in NMDAR1 protein expression, improve rat learning and memory, and safeguard nervous system function.

## Conclusion

In summary, this study demonstrated that 1% taurine treatment ameliorated cognitive impairment caused by VOCs in young rats. Taurine protects nerve injury by down-regulating GFAP, MBP and NF-L levels in serum and up-regulating BDNF protein expression in brain tissue, thus improving neurobehavioral function. Further, taurine ameliorates lipid peroxidation damage in the nervous system by preventing the VOCs-induced elevation of MDA levels in rat brain tissue. In addition, taurine can restore learning and memory ability and neurological damage in young rats exposed to VOCs by regulating imbalance of Glu/GABA system and activating the NMDA receptor. This study demonstrates taurine as a potential novel drug for the treatment of cognitive behavioral disorders especially in children.

## Data availability statement

The raw data supporting the conclusions of this article will be made available by the authors, without undue reservation.

## Ethics statement

The animal study was reviewed and approved by Laboratory Animal Management and used Committee of the Institute for Hygiene of Ordnance Industry.

## Author contributions

YG: Conceptualization, funding acquisition, methodology, and writing—original draft. CS: Supervision and writing and editing. TG: Methodology, data curation, and writing and editing. ZL: Investigation and formal analysis. ZY: Methodology and writing. HD and PF: Methodology. JG: Project administration. All authors contributed to the article and approved the submitted version.

## Funding

This work was supported by grants from the Science and Technology Plan Project of Shaanxi Province (2021JQ-934).

## Conflict of interest

The authors declare that the research was conducted in the absence of any commercial or financial relationships that could be construed as a potential conflict of interest.

## Publisher's note

All claims expressed in this article are solely those of the authors and do not necessarily represent those of their affiliated organizations, or those of the publisher, the editors and the reviewers. Any product that may be evaluated in this article, or claim that may be made by its manufacturer, is not guaranteed or endorsed by the publisher.
